# Supplementary material for: Trends in Treatment of Head and Neck Cancer in Germany: A Diagnosis-Related-Groups-Based Nationwide Analysis, 2005–2018
Source: Cancers (Basel). 2021 Dec 1;13(23):6060. doi: 10.3390/cancers13236060 (PMC8656765; doi:10.3390/cancers13236060)
Supplement: Supplementary file 1 [file cancers-13-06060-s001.zip › cancers-1474311-supplementary.pdf]

Supplementary Materials: Trends in Treatment of Head and Neck Cancer in Germany: A Diagnosis-Related-Groups-Based Nationwide Analysis, 2005–2018

Isabel Hermanns Rafat Ziadat, Peter Schlattmann and Orlando Guntinas-Lichius

Table S1. Change of operation and procedure rates for oral cavity cancer in Germany over the time from 2005 to 2018.

OPS-Code	Estimate	StdErr	ChiSq	p	RR	(95% CI)
8-52	0.0175	0.0039	19.71	<0.0001	1.09	(1.05–1.13)
8-54	0.0188	0.0049	14.92	0.0001	1.10	(1.05–1.15)
5-270	0.0081	0.0069	1.39	0.2383	1.04	(0.97–1.11)
5-271	0.0262	0.0193	1.84	0.1752	1.14	(0.94–1.38)
5-272	0.0131	0.0042	9.82	0.0017	1.07	(1.02–1.11)
5-273	-0.0077	0.0038	4.23	0.0397	0.96	(0.93 – 1.00)
5-274	-0.0206	0.0070	8.69	0.0032	0.90	(0.84–0.97)
5-275	-0.0084	0.0072	1.35	0.2449	0.96	(0.89–1.03)
5-277	0.0113	0.0071	2.52	0.1126	1.06	(0.99–1.13)
5-278	0.0412	0.0057	51.75	<0.0001	1.23	(1.16–1.30)
5-401	0.0005	0.0051	0.01	0.9255	1.00	(0.95–1.05)
5-403	0.0172	0.0028	38.29	<0.0001	1.09	(1.06–1.12)

OPS = Operation and Procedure Classification System; StdErr = standard error; ChiSq = chi-square distribution; CI = confidence interval; 8-52 = radiotherapy; 8-54 = chemotherapy/immunotherapy; 5-270 = maxillofacial incision and drainage; 5-271 = incision of hard and soft palate; 5-272 = excision and destruction of diseased hard and soft palate; 5-273 = incision, excision and destruction in the oral cavity; 5-274 = floor of the mouth reconstruction; 5-275 = palatoplasty; 5-277 = resection of the floor of the mouth and reconstruction; 5-278 = resection of the cheek and reconstruction; 5-401 = excision of neck lymph nodes and lymph vessels; 5-403 = radical neck dissection

Table S2. Change of operation and procedure rates for oropharyngeal cancer in Germany over the time from 2005 to 2018.

OPS-Code	Estimate	StdErr	ChiSq	p	RR	(95% CI)
8-52	0.0156	0.0057	7.43	0.0064	1.08	(1.02–1.14)
8-54	0.0147	0.0058	6.42	0.0113	1.08	(1.02–1.14)
5-290	-0.0482	0.0087	30.87	<0.0001	0.79	(0.72–0.86)
5-292	-0.0571	0.0124	21.27	<0.0001	0.75	(0.67–0.85)
5-293	-0.0799	0.0121	43.51	<0.0001	0.67	(0.60–0.76)
5-294	0.0157	0.0105	2.22	0.1361	1.08	(0.98–1.20)
5-295	0.0150	0.0044	11.55	0.0007	1.08	(1.03–1.13)
5-296	0.0569	0.0150	14.39	0.0001	1.33	(1.15–1.54)
5-401	0.0128	0.0068	3.52	0.0606	1.07	(1.00–1.14)
5-403	-0.0036	0.0033	1.20	0.2736	0.98	(0.95–1.01)

OPS = Operation and Procedure Classification System; StdErr = standard error; ChiSq = chi-square distribution; CI = confidence interval; 8-52 = radiotherapy; 8-54 = chemotherapy/immunotherapy; 5-290 = pharyngotomy; 5-292 = excision and destruction of diseased pharyngeal tissue; 5-293 = pharyngoplasty; 5-294 = other reconstruction of the pharynx; 5-295 = partial resection of the pharynx; 5-296 = radical resection of the pharynx; 5-401 = excision of neck lymph nodes and lymph vessels; 5-403 = radical neck dissection

Table S3. Change of operation and procedure rates for hypopharyngeal cancer in Germany over the time from 2005 to 2018.

OPS-Code	Estimate	StdErr	ChiSq	p	RR	(95% CI)
8-52	-0.0133	0.0062	4.58	0.0323	0.94	(0.88–0.99)
8-54	-0.0139	0.0053	6.84	0.0089	0.93	(0.89–0.98)
5-290	-0.0903	0.0086	111.02	<0.0001	0.64	(0.59–0.69)
5-292	-0.0591	0.0088	45.16	<0.0001	0.74	(0.68–0.81)
5-293	-0.0973	0.0155	39.29	<0.0001	0.61	(0.53–0.72)
5-294	-0.0054	0.0076	0.49	0.4827	0.97	(0.90–1.05)
5-295	-0.0244	0.0087	7.86	0.0051	0.89	(0.81–0.96)
5-296	0.0335	0.0120	7.86	0.0051	1.18	(1.05–1.33)
5-300	-0.0584	0.0055	111.94	<0.0001	0.75	(0.71–0.79)
5-301	-0.0656	0.0115	32.30	<0.0001	0.72	(0.64–0.81)
5-302	-0.0621	0.0060	106.53	<0.0001	0.73	(0.69–0.78)
5-303	-0.0126	0.0047	7.15	0.0075	0.94	(0.90–0.98)
5-401	-0.0280	0.0106	7.04	0.0080	0.87	(0.78–0.96)
5-403	-0.0307	0.0044	48.07	<0.0001	0.86	(0.82–0.90)

OPS = Operation and Procedure Classification System; StdErr = standard error; ChiSq = chi-square distribution; CI = confidence interval; 8-52 = radiotherapy; 8-54 = chemotherapy/immunotherapy; 5-290 = pharyngotomy; 5-292 = excision and destruction of diseased pharyngeal tissue; 5-293 = pharyngoplasty; 5-294 = other reconstruction of the pharynx; 5-295 = partial resection of the pharynx; 5-296 = radical resection of the pharynx; 5-300 = excision and destruction of diseased tissue of the larynx; 5-301 = hemilaryngectomy; 5-302 = other partial laryngectomy;: 5-303 =laryngectomy; 5-401 = excision of neck lymph nodes and lymph vessels; 5-403 = radical neck dissection

Table S4. Change of operation and procedure rates for laryngeal cancer in Germany over the time from 2005 to 2018.

OPS-Code	Estimate	StdErr	ChiSq	p	RR	(95% CI)
8-52	0.0063	0.0047	1.81	0.1779	1.03	(0.99–1.08)
8-54	0.0030	0.0039	0.62	0.4304	1.02	(0.98–1.05)
5-300	-0.0244	0.0023	115.99	<0.0001	0.89	(0.87–0.91)
5-301	-0.0483	0.0060	64.83	<0.0001	0.79	(0.74–0.83)
5-302	-0.0095	0.0023	17.47	<0.0001	0.95	(0.93–0.98)
5-303	-0.0096	0.0021	19.99	<0.0001	0.95	(0.93–0.97)
5-401	-0.0352	0.0093	14.33	0.0002	0.84	(0.77–0.92)
5-403	-0.0198	0.0018	125.34	<0.0001	0.91	(0.89–0.92)

OPS = Operation and Procedure Classification System; StdErr = standard error; ChiSq = chi-square distribution; CI = confidence interval; 8-52 = radiotherapy; 8-54 = chemotherapy/immunotherapy; 5-300 = excision and destruction of diseased tissue of the larynx; 5-301 = hemilaryngectomy; 5-302 = other partial laryngectomy; 5-303 = laryngectomy; 5-401 = excision of neck lymph nodes and lymph vessels; 5-403 = radical neck dissection

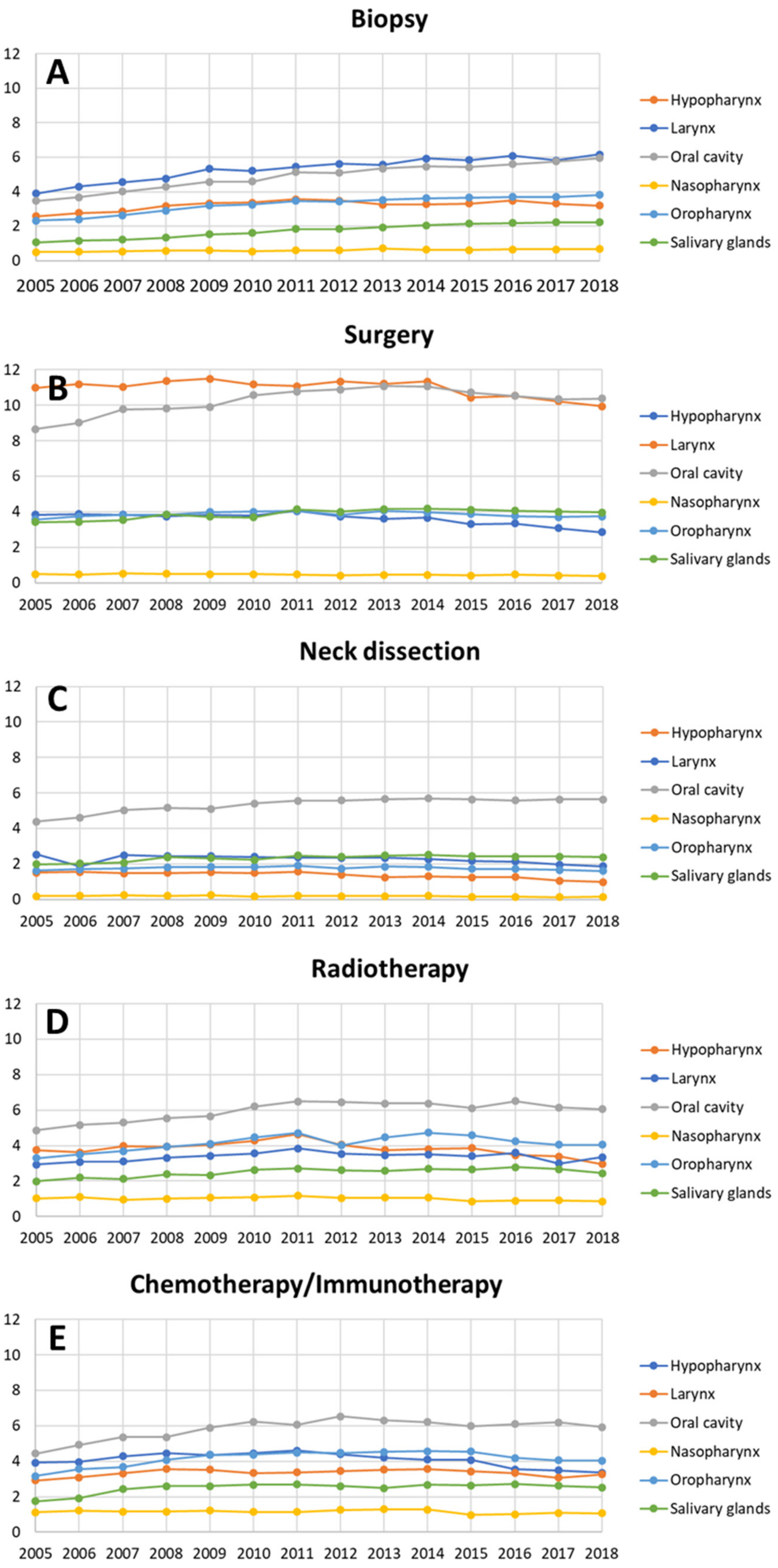

Figure S1. Annual treatment rates per 100,000 population for head and neck cancer from 2005 to 2018. A: biopsy rates; B: surgery of the primary tumor rates; C: neck dissection rates; D: radiotherapy rates, and E: chemotherapy/immunotherapy rates.
